# Eyes and movement differences in unconscious state during microscopic procedures

**DOI:** 10.1038/s41598-025-88647-4

**Published:** 2025-02-25

**Authors:** Akiko Fujita, Shintaro Oyama, Masahiro Tatebe, Shingo Shimoda, Katsuhiro Tokutake, Michiro Yamamoto, Hitoshi Hirata

**Affiliations:** 1 Department of Orthopaedics, Nagoya memorial hospital, Nagoya, 468-8520 Aichi Japan; 2 Innovative Research Center for Preventive Medical Engineering, Nagoya University, Nagoya, 464-8601 Aichi Japan; 3 Department of Orthopaedics, Anjo Kosei Hospital, Anjo, 446-8602 Aichi Japan; 4 Nagoya University Graduate school of Medicine, Nagoya, 466-8560 Aichi Japan; 5 Department of Human Enhancement & Hand Surgery, Nagoya University Graduate school of Medicine, Nagoya, 466-8560 Aichi Japan

**Keywords:** Microsurgery, Microscopic procedure, Eye movements, Eye tracker, Surface electromyography, Biological techniques, Biophysics, Computational biology and bioinformatics, Neuroscience, Medical research, Neurology, Engineering

## Abstract

Microsurgery is one of the techniques that is increasingly being adopted in many surgical fields. However, the acquisition and transfer of microsurgical skills primarily depend on experience. Additionally, opportunities to improve microsurgical skills are limited and a uniform evaluation system is lacking. Therefore, the aim of this study was to understand the physical characteristics of experienced and novice surgeons and to propose efficient training and evaluation methods from an educational perspective. In this study, nine hand surgeons and six orthopedic surgeons were included in expert group E and novice group N, respectively. An eye tracker and surface electromyography were used. They were asked to perform the suturing procedure under the same conditions. The viewpoint distribution area was larger in group N than in group E (*p* < 0.01). In group E, the pupil diameter increased only in a limited phase. The standard deviation of the distance between gaze and hand movements was smaller in group E, especially for gaze. Group E used the synergy of the same muscles to create movement. This study showed that there are differences in eye movements and unconscious body control during suturing techniques under the microscope between experienced users and novices.

## Introduction

Microsurgery is increasingly being adopted across various surgical fields. However, the acquisition and transfer of microsurgical skills largely depend on experience. Their opportunities to acquire skills are limited compared to athletes^1^. Appropriate education and training are essential for acquiring skills in microsurgery, as with other surgical procedures. Microsurgical techniques, which are widely used to reattach amputated limbs and various skin flaps, involve vascular anastomoses and nerve sutures in the extremities; these are often less than 1 mm in diameter and require the use of an operating microscope. In general, learning surgical techniques involves apprenticeship. However, in cases of finger amputation and severe limb trauma—where microsurgery is essential—the target tissue can be soft and deformed, making it difficult to quantify. Additionally, the timing for these surgeries is often urgent, precluding the use of various simulation technologies and intraoperative navigation that are becoming more common in other surgical training contexts.

Recently, various simulation and augmented reality (AR) technologies have been enhancing the efficiency of learning common surgical procedures. In Europe, microscopes that incorporate these technologies are available, although they are not yet widespread.

In daily activities like walking or reaching, it is possible to respond unconsciously to slight environmental changes or uncertainties. A seminal experiment highlighting this unconscious adaptation involves the treadmill walking test in decerebrated cats^2^. These animals were shown to maintain locomotion on a treadmill and adjust their gait patterns according to the treadmill’s speed, illustrating an implicit adaptive response to environmental dynamics. Furthermore, the anticipatory nature of motion control plays a pivotal role in unconscious adaptation. Experimental^3,4^ and theoretical^5^ evidence suggests that human movements are fine-tuned to align with environmental conditions prior to the conscious recognition of such changes. This principle of unconscious movement adaptation has been applied to the control of arm prostheses, enhancing their effective use in real-world settings^6^. Surgical experience involves the unconscious processing of surgical procedures. And surgeons must be acutely aware of their fingertips, the tips of their instruments, and even the surgical site itself. “Responsiveness” to unquantifiable uncertainties, which involves unconscious processing, is essential. This research was initiated based on the premise that it might be possible to visualize this phenomenon.

The gaze analysis technique used in this study is non-invasive. It has started to be applied across various fields because it can reflect subtle unconscious changes in brain activity through the analysis of eye movement patterns, their distribution, and pupil diameter changes. There are reports suggesting that gaze analysis is beneficial for understanding decision-making processes in the manufacturing and distribution sectors^7^. In the medical field, numerous studies have utilized this method in laparoscopic surgery^8,9^, indicating its reliability as quantitative and objective data. This method may also enhance surgical training to improve performance^10^. There is a common saying that “the eyes are as expressive as the mouth,” suggesting that by observing eye movements, one can understand latent cognitive behaviors not expressed in words. Eye movements are indicative of brain function, and it has been reported that saccade movements can help detect diseases^11^.

As for electromyograms, the use of surface electromyograms allows non-invasive and continuous data collection. The experimental results demonstrated a positive correlation between manipulation performance and maneuvering experience^12^. Additionally, this method helps to depict unconscious cognitive behavioral models^13^.

## Results

Groups E and N required average suture times of 15 min and 17 min, respectively. Four patients in group N failed to suture six stitches within 20 min. Furthermore, the mean times for a single-stitch suture were 2.5 min for group E and 4.1 min for group N; this difference was not statistically significant (*P* > 0.05).

**Fig. 1:**
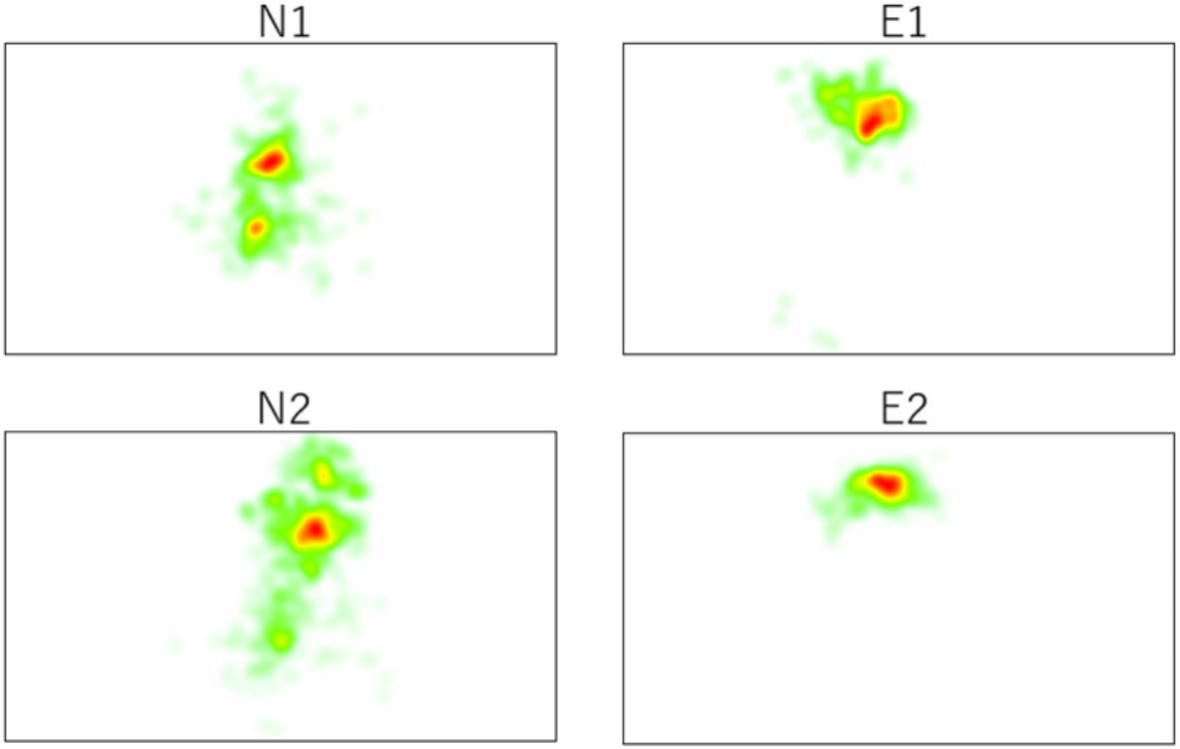
Gaze heatmap.

A heat map of the distribution of eye gazing is presented in Fig. 1.

**Fig. 2:**
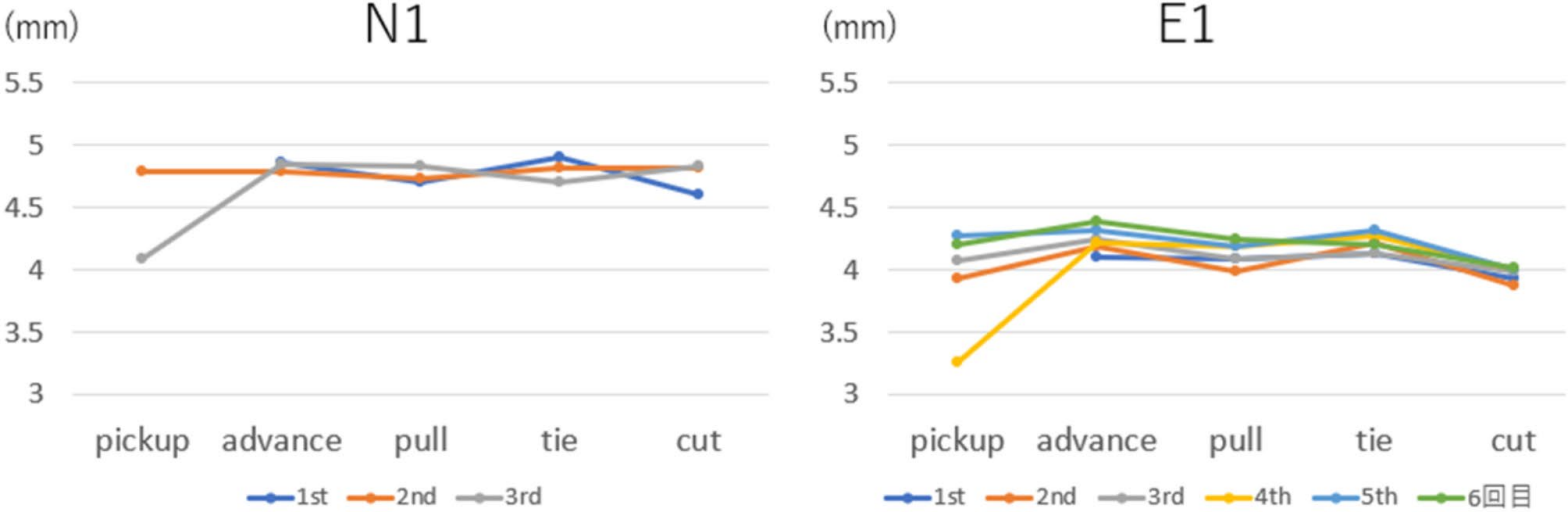
Pupil diameter transition of subjects N1 and E1.

The suture work area of group E on the 3D display can be seen as a concentrated red area in the gaze heatmap (see the right column in Fig. 2). The gaze of group N (left column, Fig. 2) was broadly distributed, possibly because the participants spent time looking at their hands. A comparison of this heatmap with color-weighted averages revealed that the area of the N group (*p* < 0.01) was predominantly larger than that of the E group.

**Fig. 3:**
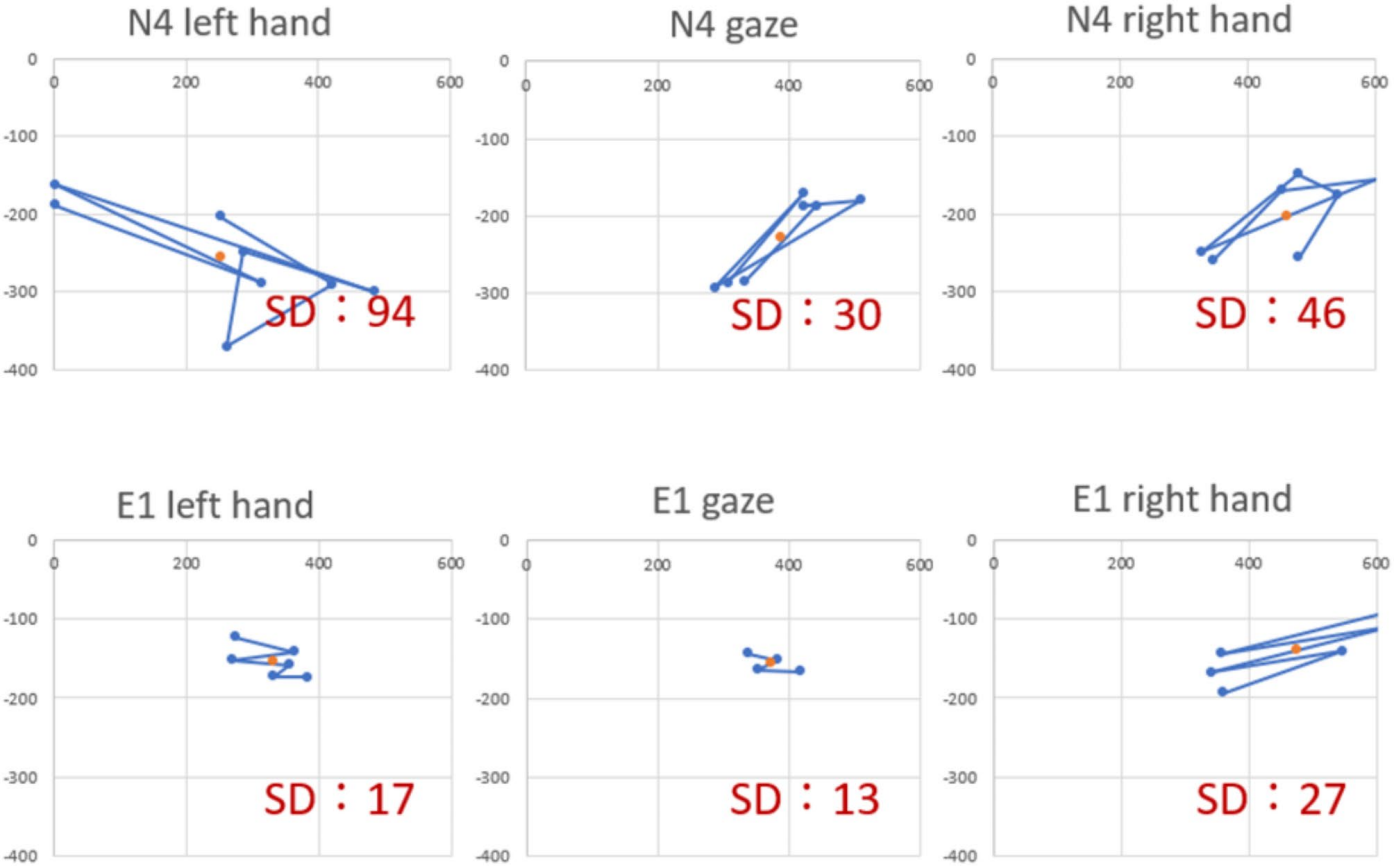
Coordinate changes and standard deviations of eye and hand movements. The red dot shows the center of gravity.

Graphs of the pupil diameter are shown in Fig. 3. The pupil diameter transition for each phase in groups E and N showed a line that was reproducible over several phases. The pupil diameter increased during the advancing and tying phases of the needle, especially in group E.

We recorded the suture movements individually on video and followed the trajectory of the gaze.

**Fig. 4:**
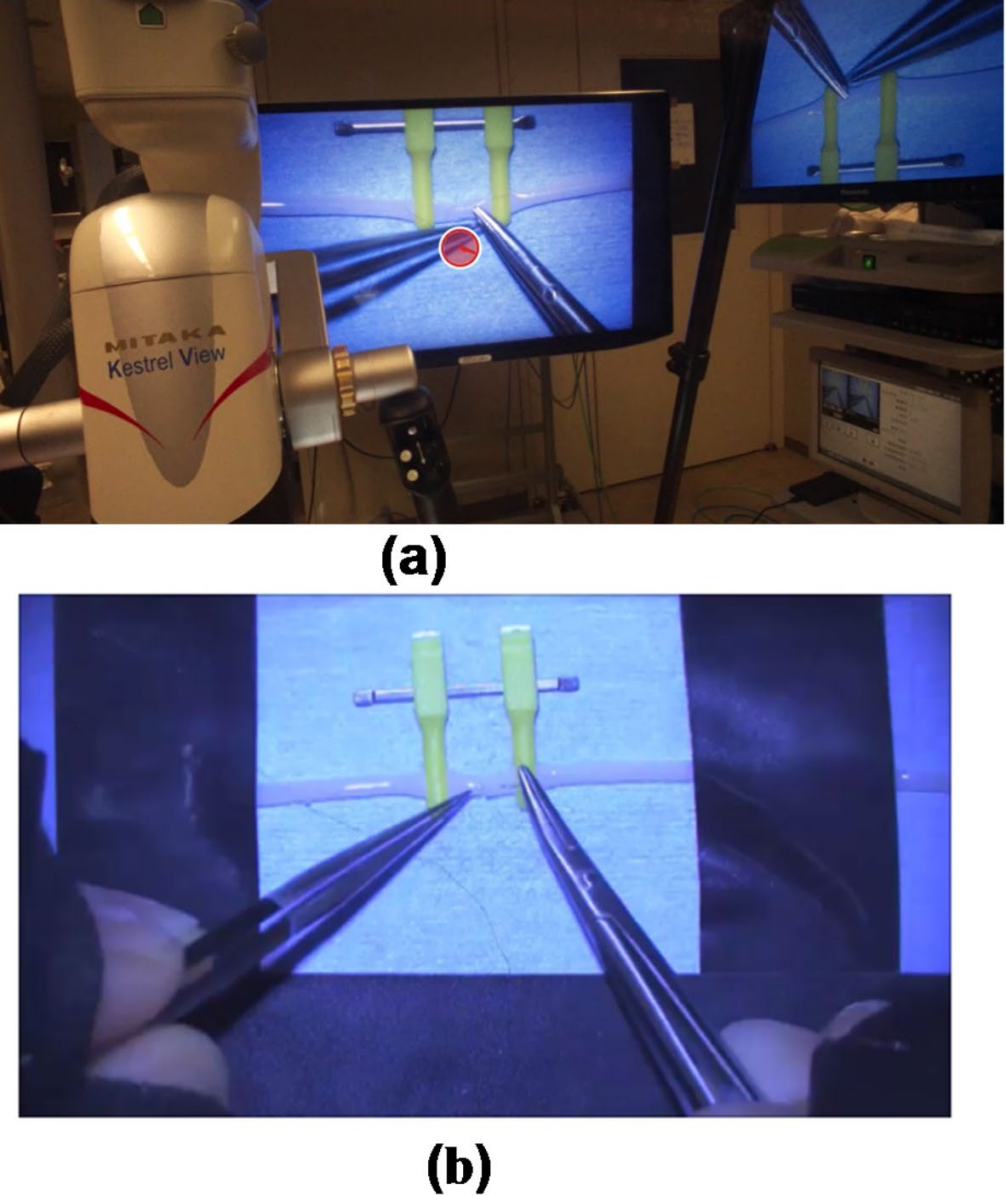
(**a**) Eye tracker video recording. The red circle in the center of the monitor shows the area being watched. (**b**) Animation of a microscope. We used MM51/YOH (Mitaka Kohki Co., Ltd., Tokyo, Japan) exoscope equipped with a 55-inch full high-definition 3D monitor. The 3D microscope had a magnification of 110×, and its working distance was 200–1,000 mm.

First, we found that the gaze of group E remained concentrated around the suture area and close to the knot throughout the series of operations without showing significant movement (Fig. 4).

We then examined the movement of forceps in the microscopic video. Group E did not waste much time (see lower panel of Fig. 4). Furthermore, the left hand barely moved, while the right hand moved to tie the thread. The tying thread of group N was located far. Hence, group N showed common movement for picking up the tying thread. The trajectory of the right hand was not stable, and the left hand pulling the thread also moved significantly.

We marked the red dots as the centers of gravity of the gaze; we also marked each hand movement for one suture. Furthermore, we calculated the distance between each point and the center of gravity. Additionally, we determined the variance. The standard deviations of group E were smaller than those of group N, especially for gaze.

The SSI was close to one in the expert group, and the same muscle synergy was used to generate the movement with six sutures. On the other hand, beginners had an SSI close to zero and generated similar movements using completely different muscle synergies each time.

## Discussion

The relationship between eye movement and proficiency has been reported in various fields. The time required for training for safety quality checks in corporate manufacturing lines and construction sites can be significantly reduced by providing feedback on eye movement results^14^. In the field of medicine, compared to novices, skilled operators perform tasks during head and neck 3D endoscopic surgery within a narrower range of starting times and places, possibly because the conversion of interpretation from 2D to 3D is empirically unnecessary for skilled operators^15^.

Several studies on gaze analysis in the field of laparoscopy have also been reported, with some reporting that evaluations using gaze analysis are more sensitive to expertise.

Variations in pupil diameter and workload have also been reported^16–19^. Our experiment showed that both groups, E and N, had increased pupil diameters in the two needle and ligature tasks, indicating the involvement of stress in that task. However, stress does exist even when the subjects concentrate on the task. The smaller fluctuations in pupil diameter of Group N than those of Group E during the task suggests that Group N constantly felt stress throughout the test and could not concentrate, when necessary, whereas Group E had a moderate level of stress and could concentrate sufficiently.

Several studies have examined the relationship between hand motions and skills in laparoscopy and arthroscopy. Yamamoto et al.^20^ used a 3D position tracker to acquire the arthroscopic motion in skilled and unskilled elbow arthroscopists and found that the amount of rotation in the y-axis direction was predominantly greater in skilled operators than in unskilled operators. In addition, Ignacio et al.^21^ showed validity in assessing proficiency in laparoscopic surgery with the time, distance, and depth of movement of manipulating instruments. Furthermore, Grober et al.^22^ studied hand motion distance with sensors on hands in microscopic techniques; they reported that this distance was objective and sensitive.

However, the hand barely moves in microsurgery; hence, the dependence is often on the movement of the fingertips. This study manually extracted and analyzed the coordinates from recorded gaze videos owing to the lack of sensor or image recognition software to capture minute movements of the fingertips and the tips of the forceps.

The predominance of a larger gaze distribution area of group N suggests a more focused approach of group E.

Changes in pupillary diameter showed the presence of a workload during needle movement and ligation. The work stress of group E increased gradually, whereas group N remained stressed throughout the task.

The forceps and eye movements during ligature demonstrated that group E showed fewer hand and eye movements centered on the knot where the gaze was focused. We believe that group E included experiential anticipatory movements.

The long movement distance of the forceps in group N may be a point to consider during training, where trainees can be instructing to be mindful of a compact movement in the next action of picking up the thread.

In surface myoelectricity, from the results of the SSI, it was found that experts with an SSI close to one generate similar movements using the same muscle synergy every time. As experts are already aware of the needle movement during the suture procedure “advance,” they can reduce the dimensionality of the difference in the situation and environment and can respond to it “unconsciously.” Novices use completely different muscle synergies to generate similar movements each time; hence, it is thought that beginners may have not yet learned how to move needles, and that they respond to differences in situations and environments through “conscious” thinking.

## Methods

### Participants

The procedures of this study were carried out in accordance with approved guidelines. This study was approved by the Clinical Research Board (CRB) of the Nagoya University Hospital (2022-0078). Written informed consent was obtained from all subjects and all experiments were conducted in accordance with the Declaration of Helsinki. Nine hand surgeons and six orthopedic surgery residents comprised the expert group E and the novice group N, respectively. The characteristics of each group are presented in Table 1. Six senior residents lacked experience in microsurgery; however, one had assisted in microsurgical procedures.

**Table 1:** Details of the subjects.

Group	Novice	Experts
Gender	1 female, 5 males	9 males
Ave. age (years)	30	42
Ave. surgical practice (years)	3.5	14.5
Surgeon’s experience (person)	0	9
Ave. microsurgical practice (years)	4	14
Dominant hand	6 right	6 right, 3 left
Dominant eye	6 right	7 right, 2 left

### Equipments and procedures

The entire process was performed in a simulated operating room set up in the laboratory with appropriate voltage conditions, lighting, and other operating environment requirements, in accordance with the equipment standards and manufacturer’s recommendations. We employed an external video microscope (MM51/YOH, Mitaka Kohki Co., Ltd.) for heads-up surgery along with a Tobii Glass 2 eye tracker (Tobii Technology, Inc.) to record eye gaze and pupil diameter. Postures were captured from three directions using three video cameras (HC-WX1M/WZX1M, Panasonic). Surface electromyogram measurements were taken using FreeEGM1000 (BTS Bioengineering), with electrodes attached to the following muscles: Hand: first dorsal interosseous; Arms: flexor carpi, extensor carpi, biceps, triceps, anterior deltoid, posterior deltoid; Trunk: upper trapezius, pectoralis major, latissimus dorsi. A total of 20 channels of electrodes were attached bilaterally.

A 1.5-mm diameter silicone artificial blood vessel (Astec Corporation, GF15U) was utilized for the suturing techniques. Participants donned a wearable eye-tracking device (Tobii Pro Glass 2) equipped with polarized lenses for the 3D display. Under the video microscope, participants were instructed to suture the severed end of the silicone artificial blood vessel with six stitches using a 10 − 0 nylon thread (ETHICON Corporation, ETHILON 10 − 0 circular needle 3 mm 3/8 c) within 20 min. The average time required to suture one stitch was calculated by dividing the total suture time by the number of sutures completed.

### Analysis

We utilized Tobii Labo analysis software in conjunction with the Tobii Pro Glass 2 to analyze eye gaze distribution, record changes in pupil diameter, and monitor hand movements beyond the line of sight, comparing groups E and N. The suturing process was segmented into five distinct steps: pick-up, advance, pull, tie, and cut. For each step, pupil diameter, eye movement, and forceps tip coordinates were recorded and subsequently analyzed.

In the surface EMG measurements, muscle synergy was calculated at each “advance” stage, and the muscle synergy stability index (SSI) was employed to evaluate the proficiency of experts and novices. SSI is an index that assesses whether movements are produced using stable muscle synergies. It ranges from 0 to 1, where values closer to 1 indicate consistent use of the same muscle synergies to generate movements, while values closer to 0 denote less consistency. This metric was determined by segmenting muscle activity during six “advance” tasks, analyzing it with a non-negative matrix, assessing the synergy correlation, and computing the SSI^8^.

## Conclusions

The results of this study showed differences in gaze distribution, pupil diameter, movement of forceps, and body control during suturing techniques under a microscope between experienced and novice users. These differences were due to unconscious motions. Based on the results of the experiments conducted, we are currently examining which scores improve with repeated practice. In the future, the results of the study must be validated in terms of education and transfer of skills. The setting of practice times for the most stressful tasks and the items that can be improved by these practices should also be investigated based on the trends observed in the present study.

## Data Availability

All data generated are included in the paper.

## References

[1] Margaret, C., Carol-Anne, M., Shelly, L. & Tulin, C. What surgeons can learn from athletes: Mental Practice in sports and surgery. *J. Surg. Educ.* **71** (2), 262–269 (2014). 24602719 10.1016/j.jsurg.2013.07.002

[2] Shik, M. & Orlovsky, G. Neurophysiology of locomotor automatism. *Physiol. Rev.* **56** (3), 465–501 (1976). 778867 10.1152/physrev.1976.56.3.465

[3] Massion, J. ,.Movement, posture and equilibrium: interaction and coordination. *Prog Neurobiol.* **38**, 35–56 (1992). 1736324 10.1016/0301-0082(92)90034-c

[4] Patla, A. Strategies for dynamic stability during adaptive human locomotion. *IEEE Eng. Med. Biol. Mag*. **22**, 48–52 (2003). 12733458 10.1109/memb.2003.1195695

[5] Huxham, F., Goldie, P. & Patla, A. Theoretical considerations in balance assessment. *Aust J. Physiother*. **47**, 89–100 (2001). 11552864 10.1016/s0004-9514(14)60300-7

[6] Oyama, S. et al. Biomechanical Reconstruction using the Tacit Learning System: Intuitive Control of Prosthetic Hand Rotation. *Front. Neurorobot*. **10**, 19 (2016). 27965567 10.3389/fnbot.2016.00019 PMC5126704

[7] Ting, Z., Christoph, H. G. & Eric, H. G. Opportunities for using eye tracking technology in manufacturing and logistics: systematic literature review and research agenda. *Computers Industrial Engineeering*. **171**, 108444 (2022).

[8] Chuhao, W. et al. Eye-Tracking metrics predict perceived workload in robotic surgical skills training. *Hum. Factors*. **62**, 1365–1386 (2020). 31560573 10.1177/0018720819874544 PMC7672675

[9] Jian-Yang, Z., Sheng-Lin, L., Qing-Min, F., Jia-Qi, G. & Qiang, Z. Correlative evaluation of mental and physical workload of laparoscopic surgeons based on surface electromyography and eye-tracking signals. *Sci. Rep.* **7**, 11095 (2017). 28894216 10.1038/s41598-017-11584-4 PMC5594030

[10] Tony, T. et al. Eye tracking for skills assessment and training: a systematic review. *J. Surg. Res.* **191**, 169–178 (2014). 24881471 10.1016/j.jss.2014.04.032

[11] Johnson, B. P., Rinehart, N. J., White, O., Millist, L. & Fielding, J. Saccade adaptation in autism and asperger’s disorder. *Neuroscience* **243**, 76–87 (2013). 23562581 10.1016/j.neuroscience.2013.03.051

[12] Liming, C. et al. Muscle synergies in joystick manipulation. *Front. Physiol.* **14**, 1–14 (2023). 10.3389/fphys.2023.1282295 PMC10611508 37900948

[13] Tytus, W., Fady, A., Shingo, S. & Hidenori, K. Muscle synergy stability and human balance maintenance. *J. Neuroeng. Rehabil.*, **11**,129 (2014). 10.1186/1743-0003-11-129 PMC4161771 25174281

[14] Morita, J., Fujimoto, N. & Yanagita, K. Approach to the transfer of quality control skills at the construction site using eye-gazing data. In *The 32nd Annual Conference of the Japanese Society for Artificial Intelligence* (2018).

[15] Niederhauser, L., Fink, R., Mast, F., Caversaccio, M. & Anschuetz, L. Video learning of surgical procedures: a randomized comparison of microscopic, 2- and 3-dimensional endoscopic ear surgery techniques. *Otology Neurotology*. **43**, 746–752 (2019). 10.1097/MAO.0000000000003550 35763494

[16] Beatty, J. Task-evoked pupillary responses, processing load, and the structure of processing resources. *Psychol. Bull.* **91**, 276–292 (1982). 7071262

[17] Beatty, J. & Kahneman, D. Pupillary changes in two memory tasks. *Psychon Sci.* **5**, 371–372 (1966).

[18] Granholm, E. & Steinhauer, S. R. Pupillometric measures of cognitive and emotional processes. *Int. J. Psychophysiol.* **52**, 1–6 (2004). 15003368 10.1016/j.ijpsycho.2003.12.001

[19] Menekse, D., Cagiltay, N., Ozcelik, E. & Maras, H. Insights from pupil size to mental workload of surgical residents: feasibility of an educational computer-based surgical simulation environment (ECE) considering the hand condition. *Surg. Innov.* **25**, 616–624 (2018). 30205777 10.1177/1553350618800078

[20] Yamamoto, M. et al. Experimental pilot study for augmented reality-enhanced elbow arthroscopy. *Sci. Rep.* **11**, 4650 (2021). 33633227 10.1038/s41598-021-84062-7 PMC7907139

[21] Ignacio, O. et al. Relevance of motion-related Assessment Metrics in laparoscopic surgery. *Surg. Innov.* **20** (3), 299–312 (2013). 22983805 10.1177/1553350612459808

[22] Grober, E. et al. Validation of novel and objective measures of microsurgical skill: hand-motion analysis and stereoscopic visual acuity. *Microsurgery* **23**, 317–322 (2003). 12942521 10.1002/micr.10152

